# Supplementary material for: Flagged observation analyses as a tool for scoping and communication in integrated ecosystem assessments
Source: PLoS One. 2024 Sep 23;19(9):e0305716. doi: 10.1371/journal.pone.0305716 (PMC11419343; doi:10.1371/journal.pone.0305716)
Supplement: S1 Fig — The y-axis indicates the value subtracting mean value of yearly data from 1980 to 2020. The x-axis indicates year. The negative/positive values correspond lower/higher temperature to the mean value. (PDF) [file pone.0305716.s001.pdf]

S1 Fig

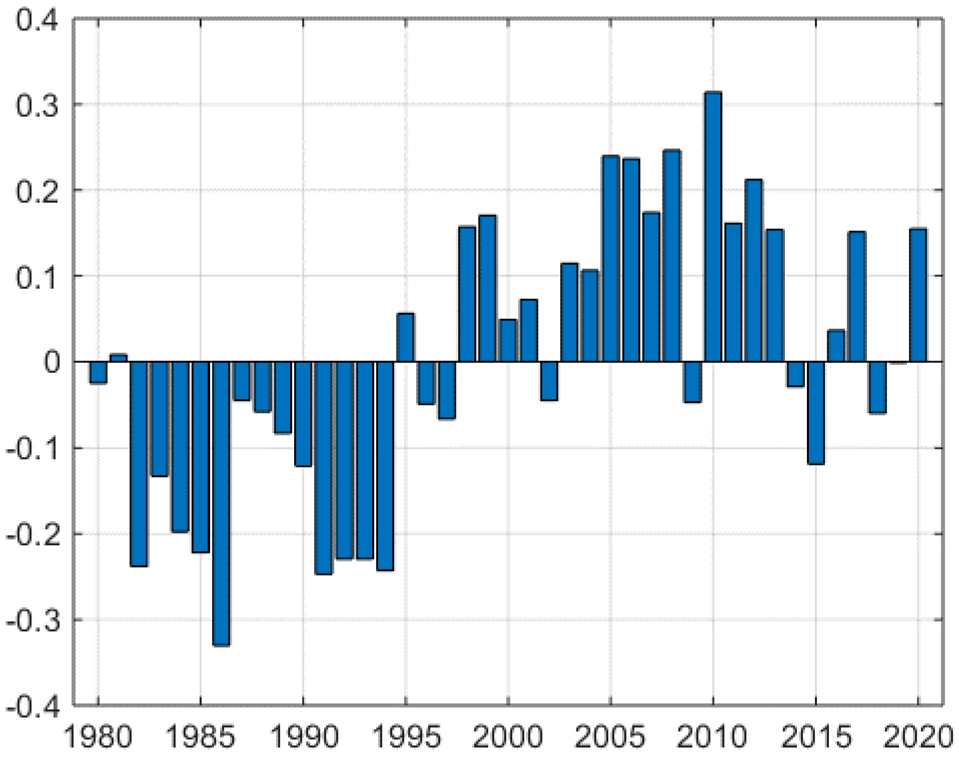

Bar plots for anomalies of AMOS's yearly data from 1980 to 2020. The y-axis indicates the value subtracting mean value of yearly data from 1980 to 2020. The x-axis indicates year. The negative/positive values correspond lower/higher temperature to the mean value.
