# Supplementary material for: Flagged observation analyses as a tool for scoping and communication in integrated ecosystem assessments
Source: PLoS One. 2024 Sep 23;19(9):e0305716. doi: 10.1371/journal.pone.0305716 (PMC11419343; doi:10.1371/journal.pone.0305716)
Supplement: S2 Fig — Monthly time series data: The estimated three-months (upper) and seven-months (lower) prediction values of sea surface temperature (blue lines) and the most recent observations for the dataset on the Atlantic Multi-decadal Oscillation from January 1980 to December 2020. The solid grey lines indicate the observations used for making the forecast values and the black points indicate the observations that were plotted for comparison with the prediction values (dotted blue line). The dotted grey line presents the prediction value Hz(n|n−1) and the solid blue lines present the smoothed trend estimates obtained by a fixed-interval smoother algorithm. The light-blue band presents the 95% FB (shown for the whole time series), the dark green band represents the 80% FB and light-green band the approximately 70% FB (the latter two shown for predicted years only).The estimated trends are not smooth as seen for data based on annual means but fluctuate periodically. This is because the monthly data includes seasonal fluctuations; Longer time series data: The estimated three-years (upper) and seven-years (lower) prediction values of sea surface temperature (blue lines) and the most recent observations for the monthly dataset on the Atlantic Multi-decadal Oscillation from 1990 to December 2020. The estimated trend pattern is smoothed, like the trend shown for the shorter time series (1980–2020) in Fig 3, and the FOs are the same as those identified using the shorter time series (Fig 3). (PDF) [file pone.0305716.s002.pdf]

S2 Fig

Monthly time series data

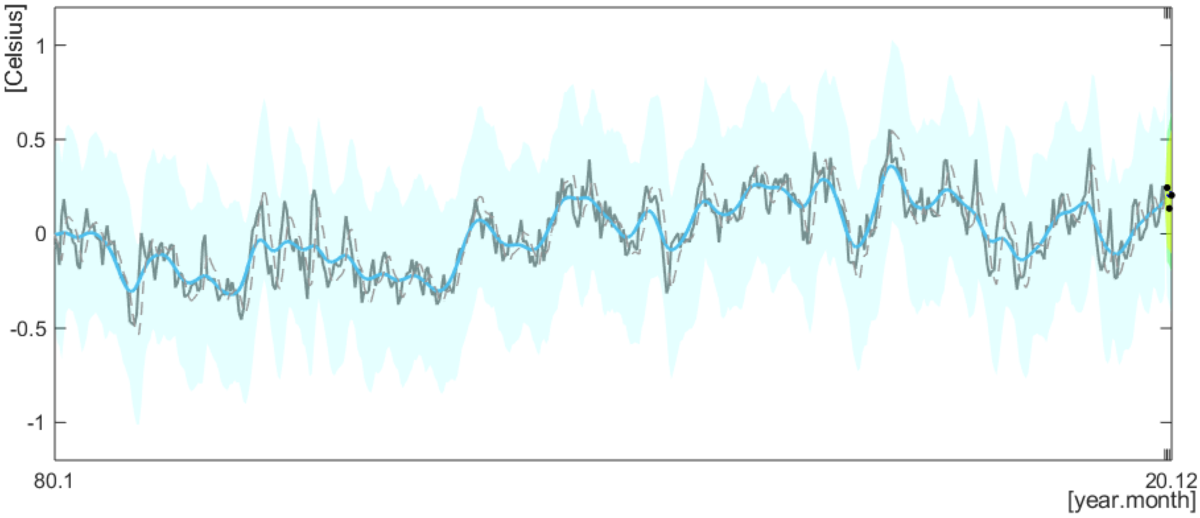

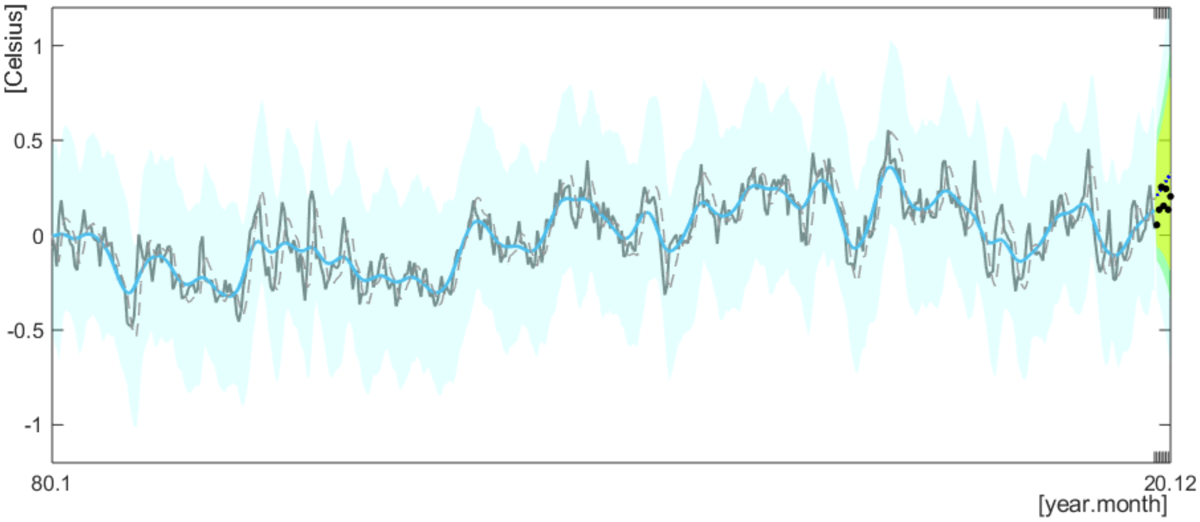

The estimated three-months (upper) and seven-months (lower) prediction values of sea surface temperature (blue lines) and the most recent observations for the dataset on the Atlantic Multi-decadal Oscillation from January 1980 to December 2020. The solid grey lines indicate the observations used for making the forecast values and the black points indicate the observations that were plotted for comparison with the prediction values (dotted blue line). The dotted grey line presents the prediction value $Hz(n|n-1)$and the solid blue lines present the smoothed trend estimates obtained by a fixed-interval smoother algorithm. The light-blue band presents the 95% FB (shown for the whole time series), the dark green band represents the 80% FB and light-green band the approximately 70% FB (the latter two shown for predicted years only).The estimated trends are not smooth as seen for data based on annual means but fluctuate periodically. This is because the monthly data includes seasonal fluctuations.

Longer annual time series data

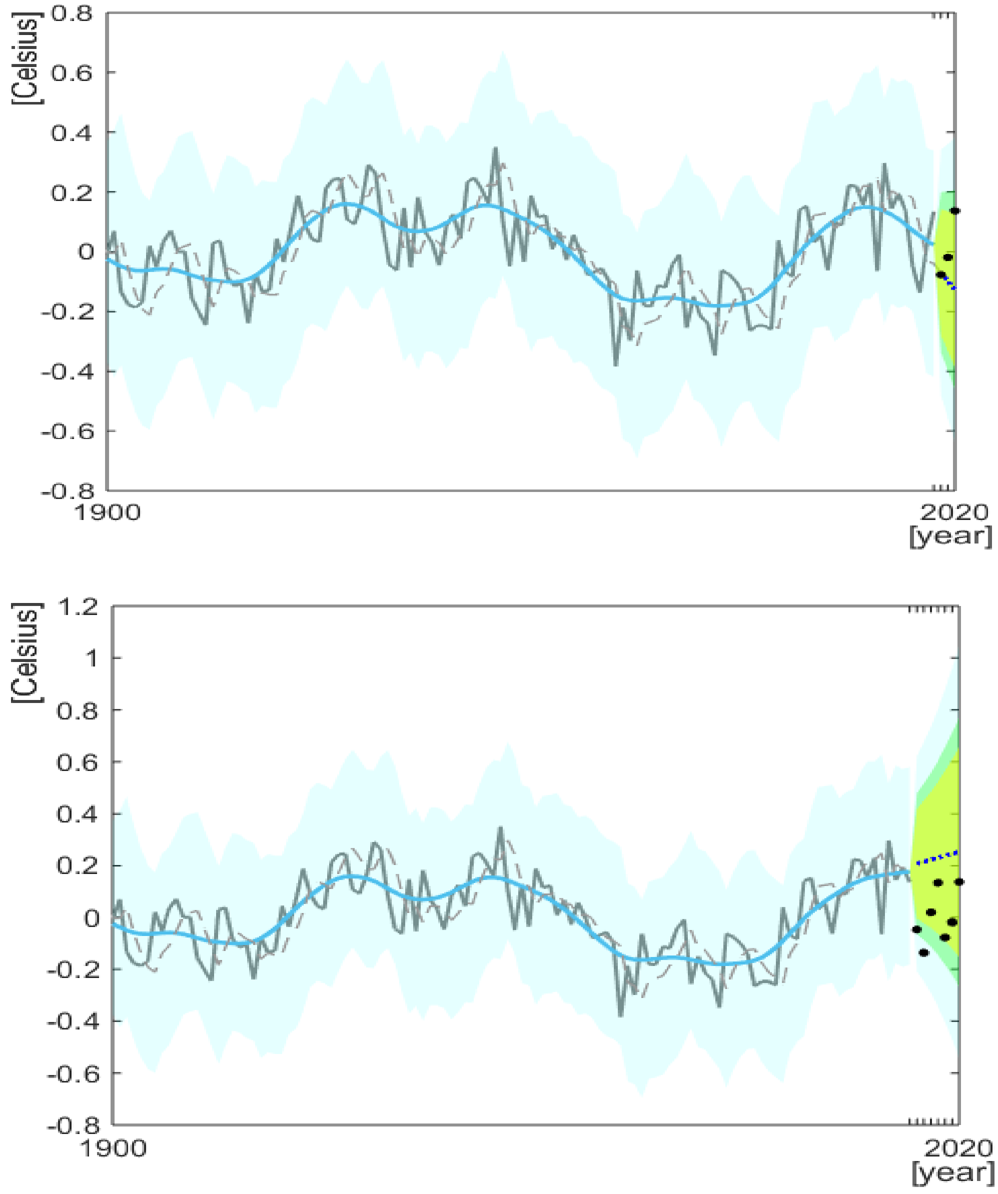

The estimated three-years (upper) and seven-years (lower) prediction values of sea surface temperature (blue lines) and the most recent observations for the monthly dataset on the Atlantic Multi-decadal Oscillation from 1990 to December 2020. The estimated trend pattern is smoothed, like the trend shown for the shorter time series (1980-2020) in Fig.2, and the FOs are the same as those identified using the shorter time series (Fig. 2).
