# Supplementary material for: Flagged observation analyses as a tool for scoping and communication in integrated ecosystem assessments
Source: PLoS One. 2024 Sep 23;19(9):e0305716. doi: 10.1371/journal.pone.0305716 (PMC11419343; doi:10.1371/journal.pone.0305716)
Supplement: S1 Table — (PDF) [file pone.0305716.s003.pdf]

S1 Table. Abbreviations used in figures and tables for data from the ICES integrated ecosystem assessment working group for the Norwegian Sea (WGINOR) and a short summary of the objective for inclusion of each of the time series in the work of the group.

Type	Abbreviation in figures and tables	Explanation of relevant data	Objective for inclusion of time series
Climate	RHC	Relative heat content in 10^8 Jm^{-2}	The time series reflects variability in water temperature, which may affect many biological processes [1-6]
	RFW	Relative freshwater content in m	There are two objectives for including this time series. Firstly, changes in freshwater content hold information about properties of inflowing water masses. This contributes to understanding of the dynamics underlying changes in the physical environment [7-9]. Secondly, climate warming may cause freshwater content in the Norwegian Sea to increase [10, 11]. This may increase water column stratification, which in turn may reduce primary production [12].
	NAO	North Atlantic Oscillation expressed as djfm	The time series is included because it holds key information about climate variability. The North Atlantic Oscillation (NAO) Index describes changes in the strength of two recurring pressure patterns in the atmosphere over the North Atlantic: a low near Iceland, and a high near the Azores Islands. The index is related to large scale patterns in climate variability, with strongly positive values linked to warm conditions across the U.S. East and Northern Europe, and cold conditions across southern Europe and strongly negative values linked to cold conditions in the U.S. East and Northern Europe, and warm conditions in Southern Europe [13].
Zooplankton	ZooB	Total mesozooplankton biomass the Norwegian Sea in in May, g m-2	Mesozooplankton is an important food source for the three large pelagic fish stocks in the Norwegian Sea [14-17] and are included to provide information about key aspects of the ecological situation for these fish stocks [18].
	MacB	Spawning stock biomass of Mackerel in million tonnes	This time series is included because mackerel supports an economically important fishery and is a functionally

Pelagic fish			important species in the Norwegian Sea ecosystem [16, 18-20]
	MacR	Recruitment of Mackerel per year class at age 0 in millions	This time series is included because recruitment is an important determinant of mackerel stock size [21]
	MacW	Weight of Mackerel at age 6 in the stock (in kg)	These time series are included because they provide information on growth of individuals, which may give information about important ecological processes, in particular intra- and interspecific competition [1, 18]
	MacL	Length of Mackerel at age 6 in cm	
	HerB	Spawning stock biomass of Norwegian spring-spawning herring in million tonnes	This time series is included because Norwegian spring-spawning herring supports an economically important fishery and is a functionally important species in the Norwegian Sea ecosystem [16, 18-20]
	HerR	Recruitment of Herring per year class at age 2 in millions	This time series is included because recruitment is an important determinant of Norwegian spring-spawning herring stock size [21, 22]
	HerW	Weight of Herring at age 6 in the stock (in kg)	These time series are included because they provide information on growth of individuals, which may give information about important ecological processes, in particular intra- and interspecific competition [1, 18]
	HerL	Length of Herring at age 6 in cm	
	BWB	Spawning stock biomass of Blue whiting in million tonnes	This time series is included because blue whiting supports an economically important fishery and is a functionally important species in the Norwegian Sea ecosystem [16, 18-20]
	BWR	Recruitment of Blue whiting per year class at age 1 in millions	This time series is included because recruitment is an important determinant of blue whiting stock size [21]
	BWW	Weight of Blue whiting age 6 in the catch (in kg)	These time series are included because they provide information on growth of individuals, which may give information about important ecological processes, in particular intra- and interspecific competition [1, 18]
	BWL	Length of Blue whiting at age 6 in cm	

References

1. Skjoldal HRe. The Norwegian Sea Ecosystem. Trondheim: Tapir Academic Press; 2004.

2. Olafsdottir AH, Utne KR, Jacobsen JA, Jansen T, Óskarsson GJ, Nøttestad L, et al. Geographical expansion of Northeast Atlantic mackerel (Scomber scombrus) in the Nordic Seas from 2007 to 2016 was primarily driven by stock size and constrained by low temperatures. Deep Sea Research Part II: Topical Studies in Oceanography. 2019;159:152-68. doi: https://doi.org/10.1016/j.dsr2.2018.05.023.
3. Nikolioudakis N, Skaug HJ, Olafsdottir AH, Jansen T, Jacobsen JA, Enberg K. Drivers of the summer-distribution of Northeast Atlantic mackerel (Scomber scombrus) in the Nordic Seas from 2011 to 2017; a Bayesian hierarchical modelling approach. . ICES Journal of Marine Science. 2018. doi: 10.1093/icesjms/fsy085.
4. Strand E, Bagøien E, Edwards M, Broms C, Klevjer T. Spatial distributions and seasonality of four Calanus species in the Northeast Atlantic. Prog Oceanogr. 2020;185:102344. doi: https://doi.org/10.1016/j.pocean.2020.102344.
5. Planque B, Fromentin JM. *Calanus* and environment in the eastern North Atlantic. I. Spatial and temporal patterns of *C. finmarchicus* and *C. helgolandicus*. Marine Ecology Progress Series. 1996;134:101-9.
6. Beaugrand G, Luczak C, Edwards M. Rapid biogeographical plankton shifts in the North Atlantic Ocean. Global Change Biology. 2009;15(7):1790-803. doi: https://doi.org/10.1111/j.1365-2486.2009.01848.x.
7. Mork KA, Skagseth Ø, Søiland H. Recent Warming and Freshening of the Norwegian Sea Observed by Argo Data. Journal of Climate. 2019;32(12):3695-705. doi: 10.1175/jcli-d-18-0591.1.
8. Skagseth Ø, Broms C, Gundersen K, Hátún H, Kristiansen I, Larsen KMH, et al. Arctic and Atlantic Waters in the Norwegian Basin, Between Year Variability and Potential Ecosystem Implications. Frontiers in Marine Science. 2022;9. doi: 10.3389/fmars.2022.831739.
9. ICES. Working Group on the Integrated Assessments of the Norwegian Sea (WGINOR, outputs from 2022 meeting).mICES Scientific Reports. 5:15. . 2023.
10. Pörtner H-O, Roberts DC, Masson-Delmotte V, Zhai P, Tignor M, Poloczanska E, et al. IPCC Special Report on the Ocean and Cryosphere in a Changing Climate. 2019.
11. Arneberg P, Siwertsson A, Husson B, Børsheim KY, Fauchald P, Høines Å, et al. Panel-based Assessment of Ecosystem Condition of the Norwegian Sea Pelagic Ecosystem. Rapport fra Havforskingen. 2023.
12. Moore JK, Fu W, Primeau F, Britten GL, Lindsay K, Long M, et al. Sustained climate warming drives declining marine biological productivity. Science. 2018;359(6380):1139-43. doi: doi:10.1126/science.aao6379.
13. NOAA. Climate Variability: North Atlantic Oscillation 2009. Available from: https://www.climate.gov/news-features/understanding-climate/climate-variability-north-atlantic-oscillation.
14. Mousing EA, Planque B, Arneberg P, Bjørdal VR, Keulder-Stenevik F, Liebig PL, et al. Quantifying diets for small pelagic fish: effects of weight versus occurrence methods and sampling effort. ICES Journal of Marine Science. 2023;80:317-28. doi: 10.1093/icesjms/fsac240.
15. Dalpadado P, Ellertsen B, Melle W, Dommasnes A. Food and feeding conditions of Norwegian spring-spawning herring (Clupea harengus) through its feeding migrations. ICES Journal of Marine Science. 2000;57(4):843-57. doi: 10.1006/jmsc.2000.0573.
16. Bachiller E, Skaret G, Nøttestad L, Slotte A. Feeding Ecology of Northeast Atlantic Mackerel, Norwegian Spring-Spawning Herring and Blue Whiting in the Norwegian Sea. PLoS One. 2016;11(2):e0149238. doi: 10.1371/journal.pone.0149238.
17. Langøy H, Nottestad L, Skaret G, Broms C, Ferno A. Overlap in distribution and diets of Atlantic mackerel (Scomber scombrus), Norwegian spring-spawning herring (Clupea harengus) and blue whiting (Micromesistius poutassou) in the Norwegian Sea during late summer. Marine Biology Research. 2012;8(5-6):442-60. doi: 10.1080/17451000.2011.642803. PubMed PMID: WOS:000303560300003.

18. Huse G, Holst JC, Utne K, Nottestad L, Melle W, Slotte A, et al. Effects of interactions between fish populations on ecosystem dynamics in the Norwegian Sea - results of the INFERNO project Preface. Marine Biology Research. 2012;8(5-6):415-9. doi: 10.1080/17451000.2011.653372. PubMed PMID: WOS:000303560300001.
19. Bachiller E, Utne KR, Jansen T, Huse G. Bioenergetics modeling of the annual consumption of zooplankton by pelagic fish feeding in the Northeast Atlantic. PLoS One. 2018;13(1):e0190345. doi: 10.1371/journal.pone.0190345.
20. Skjoldal HR, Dalpadado P, Dommasnes A. Food webs and trophic interactions. In: Skjoldal HR, editor. The Norwegian Sea ecosystem. Trondheim: Tapir Academic Press; 2004. p. 447-506.
21. ICES. Working Group on Widely Distributed Stocks (WGWIDE). ICES Scientific Reports. 1:36. . 2019.
22. Garcia T, Planque B, Arneberg P, Bogstad B, Skagseth Ø, Tiedemann M. An appraisal of the drivers of Norwegian spring-spawning herring (Clupea harengus) recruitment. Fisheries Oceanography. 2020;30(2):159-73. doi: 10.1111/fog.12510.
