# Supplementary material for: Flagged observation analyses as a tool for scoping and communication in integrated ecosystem assessments
Source: PLoS One. 2024 Sep 23;19(9):e0305716. doi: 10.1371/journal.pone.0305716 (PMC11419343; doi:10.1371/journal.pone.0305716)
Supplement: Attachment — Submitted filename: Review_PONE-D-22-30034_reviewer.pdf [file pone.0305716.s012.pdf]

REVIEW

article «Flagged observation analyses as a tool for scoping and communication in Integrated Ecosystem Assessments»

Ecosystems influence the existing conditions of human life, either improve or worsen it, and accordingly require a person's constant adaptation to such changes. Thanks to observations, it is possible to achieve the necessary predictability of ecosystems, reliably forecast and satisfy needs while under its influence. All this confirms the relevance of the article, and despite its significant advantages, it is appropriate to take into account a number of recommendations:

– the article should indicate the objectives of the observations with the overall goal of determining the scope and communications in ecosystem assessments and the expected results from such observations;

– it is worth citing the advantages and disadvantages of conducting the analysis of observations with labels, in particular, comparing them with some frequently used statistical methods;

– it is necessary to develop a set of proposals for the active implementation of the observation analysis tool with labels, addressing them to international institutions, associations of countries or others.
