# Supplementary material for: Flagged observation analyses as a tool for scoping and communication in integrated ecosystem assessments
Source: PLoS One. 2024 Sep 23;19(9):e0305716. doi: 10.1371/journal.pone.0305716 (PMC11419343; doi:10.1371/journal.pone.0305716)
Supplement: Attachment — Submitted filename: Reviewers comments and how they are dealt with.docx [file pone.0305716.s010.docx]

Dear PLOS ONE Academic Editor, Dr. Pasquale Palumbo,

This document contains our response to the comments and concerns provided to us by the Editor and two reviewers on our submitted manuscript:

Re: PONE-D-22-30034

Solvang and Arneberg: Flagged observation analyses as a tool for scoping and communication in Integrated Ecosystem Assessments

The comments from the Editor and the two reviewers are given in black – how we responded are given in red. We submit two versions of the manuscript, one showing all changes and one where all tracked changes have been accepted. The line numbers below refer to the latter.

Review Comments to the Author

Please use the space provided to explain your answers to the questions above. You may also include additional comments for the author, including concerns about dual publication, research ethics, or publication ethics. (Please upload your review as an attachment if it exceeds 20,000 characters)

Reviewer #1: I like this paper. To briefly summarize, Kato and Arneberg develop a tool based on the Kalman filter to identify anomalous observations in time series. This tool can be useful in integrated ecosystem assessments where large numbers of observations need to be considered. I do not have full overview of the literature on Integrated Ecosystem Assessments, but Kato And Arneberg provide a satisfying overview of relevant work and they presumably offer a valuable contribution.

The idea is sound and relatively straight forward: Estimate a trend with the Kalman filter and assess whether recent observations deviate sufficiently from this trend to flag them as low probability events. While the basic statistical method is developed and established in the literature and numerous textbooks, the presentation of the technicalities leaves something to desire (see list of comments below).

The approach to assess joint probabilities is non-standard (there is no established standard, as far as I know, unless all involved distributions are Gaussian). This part needs to be explained better. That 'the joint probability ... should be calculated by random variables' (lines 175-177) is cryptic. If I understand correctly, Kato and Arneberg represents the trend prediction uncertainty band by simulation. But if the distribution is assumed Gaussian, I think one can calculate the probability of a given observation from the estimated covariance. Regardless, how the simulated probability evaluation is done in a multivariate setting needs to be spelled out in detail. The relevant computer code is said to be available as supplementary material, but I only found a table of abbrevations in the supplement.

*Response: The concept is now described in the introduction. In the methods section, we have now added a description of what we want to estimate: “Thus, we want to estimate the joint probability that the location of the observations from the most recent years relative to the predicted trend could be the result of random variation. As the time series data is sampled as one sample at one time point, this should be calculated by random variables, which is generated from a Gaussian distribution. Thus, what we want to estimate, is the probability that random variation in a Gaussian distribution could have resulted in a lower estimated probability.”*

*With cross sectional data, the probability of a given observation can be calculated from estimated covariance, which corresponds to a p-value. With time series data, where each observation is from a single time point, this approach cannot be used. Therefore, an alternative approach must be applied, and here we have based this on simulations in the following way: we assume Gaussian distribution for the prediction with Hz(n+j|n) as mean and d(n+j|n) as variance. Furthermore, we conduct Monte Carlo simulations to calculate the p-value. The details are given in the methods section. See lines 185-206.*

*The computer code has now been added in supplementary files S1 and S2 with example data in supplementary file S3.*

I do not have much to say about the examples, they seem to be executed in a satisfactory fashion. But I wonder why you restrict your sample to the period 1980-2020 (see line 202) when the data series goes back to 1869.

*Response: We have mostly focused on short time series (i.e., < 50 observations), because this is typically what is available to IEA groups. In the paper, there are also examples of longer time series (e.g., for herring), and to give additional examples, we have also added analyses using a longer time series for the AMO data and also using monthly data for the AMO time series to provide an example of an even longer time series. These latter analyses have been added to the supplementary material. The main text has been revised accordingly*. *See lines 218-220, 225-228 and 248-252.*

And why do you use annual means rather than the monthly observations?

*Response: See above*

Specific comments:
- Eq. 2: Please provide the definition of the difference operator. The given expression for d=2 is not self-explanatory.

*Response:* *We have given the definition of the dth-order difference equation in lines 101-107.*

- Eq. 3: What is G? What is its interpretation?

*Response: G is integer when the trend is random walk model (d=1), and G is a vector leading that the first component corresponds to add v(n). Text describing this is added on lines 113-119.*

- Eq. 4: What is Q and what is its interpretation? On line 146, you say that Q should be 'optimal'. What is the criteria for the optimal Q? Further, the procedure involving equations 4 and 5 could be explained better. The explanation on lines 123 and forward is unclear.

*Response: We have added the explanation of Q. The criterium for the optimal Q is log-likelihood is as explained in lines143-144 and 153-157. Please note that Q-values had been set too high in the original submission. This has now been adjusted. A modification of the G vector has also been done. This has resulted in some adjustments to the results, most importantly that the decline in mackerel spawning stock biomass is now identified as a flagged observation (lines 317-322).*

- Line 137: Why can the variance of w be set to the variance of the observation? Is the variance of the observation an expression of observation uncertainty? Is the observation uncertainty known?

*Response: Because the state space model includes the observation model (the second equation) in formulae (3), the variance of the observation is an expression of observation uncertainty. The observation uncertainty is given as the variance of the observations in this study to estimate the variance of the system noise in the system equation (the first equation) in formulae (3), however; the observation uncertainty can be estimated as one parameter of the model by a numerical optimization. We have added an explanation for the flexibility to the observation noise (lines 144-146 and 156-157)*

- Line 154: Assuming Y(n+1)=Y(n), where Y(n)=(y(1), ..., y(n)) is cryptic. Presumably, Y(n+1) has the element y(n+1), which evidently is not in Y(n), so it is not clear what is assumed. It is further unclear how z(n+2|n) relates to z(n+1|n).

*Response:* *Since the future observation y(n+1) does not exist in fact, the prediction has to be estimated based on the time series Y(n) = (y(1), y(2), … , y(n)). In the sense, it is assumed that Y(n+1) = Y(n). This concept was referred in Section 9 [57]. We have added the reference in the text (line 164).*

- Eq. 7: The d-notation is already used for something else (see eq. 2), should be changed.

*Response: We have changed. (lines 177 and 179)*

- Line 203: Annual means for 1980-2020 presumably results in a time series with 41 observations.

*Response:* *text has been corrected (line 225)*

Best of luck with further work on this paper!

Thank you very much for your kind words.

Reviewer #2: This paper deals with the problem of assessing recent changes in ecosystems by suitably processing time series. The Authors propose a procedure aimed at detecting time series where the most recent observations are less expected on the basis of past data (large deviations from the prediction). The estimation of trends in a time series and the prediction on the basis of past data is obtained by exploiting a stochastic trend model and a Kalman filter/smoother/predictor algorithm. The assessment of unexpected changes is made by using estimated Forecast Bands and joint probabilities. Two examples that illustrate the proposed procedure are reported.

I would like to point out that my opinion on this paper is from the viewpoint of a person with expertise in data processing, and not on biological/ecological applications.

From my viewpoint, the methodology used by the Authors in this paper is well established (it is grounded on the works of Kalman (~1960), Akaike (~1980) and Kitagawa (~1980)). Thus, from a methodolgical point of view I don't see any contribution in this work.
In any case, the Authors should specify in what the proposed "Flagged Observation Analysis" is different from, and possibly an improvement over, the approaches in [25-28].

*Response: The approaches in [25-28] have different scopes, namely identifying change caused by specific types of dynamics and often with specific types of outcomes. Flagged Observation Analysis aims at identifying unexpected change in a way that is not specific to any type of dynamics and/or outcomes, and where the aim is to identify time series that may need to be followed up more closely in an IEA, possibly by addressing dynamics and consequences. We have added text explaining this (lines 75-78).*

A possible contribution could be in the two case studies investigated, in the field of marine ecosystems, which appear to be interesting, although I am not an expert in this field, so I cannot express a qualified opinion in this area.

*Response: Concerning the contribution of the paper, we would like to point out that the procedure for joint probability calculation is novel. In addition, the application of the methods in an IEA context in the way it is done here is also novel and should be of interest for a wide readership interested in IEAs and related frameworks.*

Below some comments on data processing aspects of the paper.

After a rather detailed presentation, in the first 6 pages of the paper, of a well established method in the field of time series analysis, the Authors present the proposed methodology for computing "joint probabilities", aimed at "Flagging" unexpected observations and assessing unexpected changes in a time series.
The procedure is outlined in the section "Assessing unexpected tendencies - joint probability for detected FO" in the lines from 180 to 188 of the paper.

However, despite it importance in the paper, the procedure is not clearly introduced and properly justified.

*Response: The need for this procedure has now been introduced and justified in the introduction. Additional description has been added to the methods section (see response to reviewer 1 above).*

The points that are obscure to me are listed below:
- it is not clear what kind of joint probability is computed in the procedure. It is understood that the computed probability concerns the observations from n+1 to n+J. However, it has not been explained what kind of probability is (is it the probability that ALL the observations from n+1 to n+J are outside a given Forecast Band? Or is the probability that AT LEAST ONE observation in the interval is outside the FB? Or what else?)

*Response: The joint probability is estimated when all observations from the recent years fall on one side of the predicted trend. The position relative to the Forecast Bands is not relevant but was erroneously described as this in the original submission. We have now corrected this and also give a more detailed explanation in the method section. Reviewer 1 raised the same issue, so please see response above.*

- in line 183 I can not understand the sentences "if y(n+j|n) is upper over FB / if y(n+j|n) is lower under FB". According to my understanding, the predicted value y(n+j|n) is in the middle of any FB, thus how can it be upper or lower w.r.t. FB?

*Response: As described above, positions relative to FBs are not relevant, but position relative to the predicted trend, and text has been corrected (line 199) .*

- there is no reported motivation on the chosen sizes of 10.000 (in the generation of random numbers, at step 1) and 1000 (for the averaging in step 3). Is there any quantitative reason for these specific choices?

*Response: Since the state space model assumes a Gaussian distribution, we use a large number as 10000 to generate the distribution to avoid bias in in the estimates. Also, to avoid biased estimation for the joint probability using one Gaussian distribution, we take the averaged joint probability. However, as you pointed, it is arbitrary. Therefore, we have not suggested a specific number of randomizations and iterations but left this open for each investigator to decide (lines 205-206).*

- since all variables are assumed Gaussian, apparently there is no need to resort to a numerical Monte Carlo procedure, since all probabilities can be accurately computed. Am I missing something?
*Response:* *This issue was also raised by reviewer 1, so please see the response above.*

Throughout the paper the Authors refer to Table 1 and Table 2. However, these tables are not included in the paper.
*Response: This must have been a technical problem with the original submission. We are sorry about this. The tables have been uploaded with the revised submission.*

A suggestion:
I see that the Atlantic Multi-Decadal Oscillation (AMO) are collected monthly, and that the Authors only employ their annual means in their procedure. In this way, the information about the yearly deviation from the mean is lost.
It would be interesting if the authors tested their procedure also on data averaged on a shorter time period (e.g., trimestral or semetral) and compared the obtained results.
*Response: We thank for this suggestion and have added analyses using monthly data (lines 225-227 and 249-252). See also response to the same issue from reviewer 1 above.*

Specific comments

-Line 124: specifying the initial variance equal to 0 is rather unusual, except for the particular case where the initial state is exactly known.
*Response: No, the initial state z(1|0) is zero and the initial variance V(1|0) is an arbitrary real number (e.g. 0.1). We have rephrased the sentence (lines 130-131).*

- Line 136: The sentence "The flexibility of the estimated trend..." the concept of flexibility should be explained

*Response: We have corrected ‘smoothness’ from ‘flexibility’ (line 143 - 144).*

- In line 141, "c", the argument of AIC(c), should be explained.

*Response: We have corrected (line 149).*

- Line 145: if the value d=2 has been chosen exploiting the Akaike criterion (as the Authors say), then the values of AIC should be shown
*Response: Since we want to estimate the trend as smooth as possible, we fixed d = 2 as indicated in reference [33]. Therefore, in this study, it is not necessary to show AIC to compare the trend model for d = 1 and the trend model for d = 2. We have added the explanation (lines 146-149).*

Typos

- Line 49. Check the sentence starting with "Her we present...", something is wrong.
*Response: Revised by adding “on” (line 50).*

- Line 121. The Kalman gain in the equations (5) should always written as K(n)

*Response: We have corrected (line 127).*

- Line 130. Replace "proceeses" with "processes"

*Response: revised (line 137).*

- Line 138. Replace "differernt" with "different"

*Response: revised (line 146).*

- Line 146: square brackets should be used for the reference [33]

*Response: revised (line 154).*
