# Supplementary material for: Flagged observation analyses as a tool for scoping and communication in integrated ecosystem assessments
Source: PLoS One. 2024 Sep 23;19(9):e0305716. doi: 10.1371/journal.pone.0305716 (PMC11419343; doi:10.1371/journal.pone.0305716)
Supplement: Attachment — Submitted filename: Reply to reviewers comments second.docx [file pone.0305716.s011.docx]

Dear PLOS ONE Straive Editorial Assistant, Bernadith Millamina,

This document contains our response to the comments and concerns provided to us by two reviewers on our submitted manuscript:

Re: PONE-D-22-30034

Solvang and Arneberg: Flagged observation analyses as a tool for scoping and communication in Integrated Ecosystem Assessments

The comments from the two reviewers are given in black – how we responded are given in red. We submit the version of the manuscript showing all tracked changes and the line number we have described are corresponding to the version with tracked changes.

Reviewer #1: Review, PONE-D-22-30034_R1
The authors have revised their manuscript. As I wrote in my previous report, I think the study is worthwhile documenting in a publication. Reviewer #2 points out that the methodology is known, but I think the application is novel and valuable. Consideration of the behavior of time series needs support by statistical tools. It now occurs to me, however, that the reliance on the normal distribution may be problematic. That the authors do so in their examples may be ok, but I recommend them to add a discussion of issues such as fat tail risk that should be accounted for in real applications. The involvement of non-Gaussian distributions would further highlight the value of the simulation framework they promote, which, as I try to argue below, is unnecessary when working with Gaussian distributions.

While we have constructed the linear state space representation by (3) to describe the proposed model (1) and (2) and the state component is estimated by linear Kalman filter, the state space model is not necessary Gaussian distribution. We have explained about the expansion to non-Gaussian model and filtering algorithms in lines 457-467.

My main concern in my previous report was that the presentation of the method needed several clarifications. The authors have tried, presumably, but there are still points that remain unclear (see below). Fortunately, the authors have now supplied their computer code, but I have not had the opportunity to study it. Maybe some of my questions could have been resolved there, but I nevertheless think the manuscript needs more work. Another concern is that the authors claims to have revised the manuscript on specific points, but they have not. In particular, I requested, related to eq. 2, the definition of the difference operator, which I admittedly likely can look up in a statistics textbook. The authors reply that the definition is given in the revised manuscript, but it is not!

We have described the definition of the difference operator in lines 111-119.is used as a difference operator $\nabla t(n)=t(n)-t(n-1)$. The notation is a general form to express the differenciation of the variabes in statistical time series analysis. We have also added the literatures to confirm the notation, e.g. pages 28, 33-34 in [29] and page 19 in [59].

Further, related to eq. 3, I asked what ‘G’ is and about its interpretation. The authors provide some presumed interpretation in their letter, claiming it is added to the manuscript. One word has been added to the manuscript (‘vectors’), which supplies nothing of the sort. Thus, one may question their motive for engaging in peer-review publication.

G is a role to operate how the system noise v(n) contributes to which component in the state vector z(n). If we assume random walk trend model, G becomes just scalar 1. If we assume the trend model with d=2, the state vector becomes $(z(n),z(n-1))^{t}$(t means transpose) and v(n) contribute to only the first component in this state. In the case, G indicates $(1,0)^{t}$, that is, $Gv(n)$in (3) is represented by$\left(\begin{matrix} 1 \\ 0 \end{matrix}\right)v(n)=\left(\begin{matrix} v(n) \\ 0 \end{matrix}\right)$ and the first component of $\left(\begin{matrix} v(n) \\ 0 \end{matrix}\right)$contributes the first component of z(n). The system model is given by $\left(\begin{matrix} z(n) \\ z(n-1) \end{matrix}\right)=\left(\begin{matrix} 2 -1 \\ 1 0 \end{matrix}\right)\left(\begin{matrix} z(n-1) \\ z(n-2) \end{matrix}\right)+\left(\begin{matrix} 1 \\ 0 \end{matrix}\right)v(n)$. We have added concrete explanation about G in lines 128-135.

My first comment regarded to method for calculating joint probabilities, and I suggested that if distributions are Gaussian, probabilities can be estimated. If I remember correctly, the joint distribution of Gaussian distributions is also Gaussian. The authors claim that with time series, 'where each observation is from a single time point', whatever that is supposed to mean (can an observation be from non-single time points?), the joint distribution stuff does not work. I am not sure, I think considering the joint distribution for consecutive instances of time series are regularly considered in time series statistics (the other reviewer makes the same point), but I am no expert, and I am not sure I contribute to clarifying the situation.

Time series data is recorded as one sample at one time point. For each time point, it is assumed that the data is modelled by the stochastic process. Therefore, ‘mean’ of time series data corresponds to the ‘trend’ component, which is not same for overall time points. In this study, we apply the trend model in (2), which is time dependent, and it is not appropriate to calculate ‘joint probability’ using each probability based on the trend component. Therefore, in this revision, we use the residual part obtained by subtracting trend from the observation, which can be assumed identical and independent Gaussian distribution. Using the residual value, the upper-tale probability (if observations consecutively appear above the trend) probability is calculated using a computational function of the Gaussian distribution function. If consequently time points are three years, we obtain three upper-tale probabilities, e.g. $P_{1},P_{2},\text{ and }P_{3}$ and the joint probability becomes $P_{1}\times P_{2}\times\text{ }P_{3}$. We have revised the calculation procedure in lines 228-259.

Notwithstanding, the authors say they assume a Gaussian distribution for predictions, with some presumably well-defined mean and variance and use Monte Carlo simulations to calculate the p(robability)-value. In their manuscript, the authors write that ‘what we want to estimate with the joint probability here, is the probability that random variation in a Gaussian distribution at each time point, could have resulted in a lower estimated joint probability’ (lines 191-193). While I find this unclear, I maintain that as long as a well-defined Gaussian distribution is assumed, simulations are not necessary to calculate probabilities. Monte Carlo simulations obviously provide a number that is close enough as long as it is appropriately carried out.

The previous description was to manually calculate upper- or lower-tale probability using random number generated by a Gaussian distribution function. As you pointed out, it is not necessary to use the procedure and we should use the computational function to directly obtain upper- or lower-tale probability of the Gaussian distribution. Furthermore, it is not related to ‘probability value’ that means ‘significant probability’ to support the null hypothesis for statistical testing. We have revised the description in lines 228-259.

With regard to the explanation of the Monte Carlo simulations, I have two comments: (i) On point 2, ‘upper over FB’ and ‘lower under FB’ remains unclear. I failed to comment specifically on this in my previous report; I just requested clarifications in general; I apologize, but these statements are cryptic. The point is further made by reviewer #2, but the statements remain in the manuscript.

We have excluded the parts related to point 2 and revised the previous procedure, which is better both statistically and computationally. Please see the revised procedures in lines 228-259 and the outline of the procedure in Fig.1b.

(ii) On point 4, the summation should run from ‘b’ equals one to M, I presume.

We have not used the notation in the revised version.

I asked about ‘Q’ in relation to eq. 4, which should be optimal with regard to, it turns out, the log-likelihood (of what?). The authors write that Q-values had been ‘set too high’ in their original analysis. But it is supposed to be optimal, not ‘set’. This is unsettling.

Q is related to $H(n) V(n|n-1) H'(n)$ used in $\Sigma(n)$ of the log-likelihood function (6). $V(n|n-1)$ in $H(n) V(n|n-1) H'(n)$ is calculated using Q in the ‘*prediction*’ procedure (4). The log-likelihood is for the model (1). We have added the explanation in lines 173-180. ‘set too high’ meant that the optimum Q value was found within the range we set higher (0.05 to 0.5), however, we re-set lower range (0.01 to 0.1) to obtain more smoothed trend.

I pointed out that ‘Y(n+1) = Y(n)’ was inconsistent. I realize it is only a notational issue, but it is confusing and looks wrong. The authors insist on sticking to the notation, presumably on grounds that it is used in some reference. Adding this reference does not add clarity to the confusing notation. I find this unfortunate. My related comment that it was unclear how z(n+2|n) relates to z(n+1|n), the authors have simply chosen to disregard.

Since the future observation y(n+1) is not observed, the calculation is formally conducted by *assuming* approximately Y(n+1)=Y(n), which was taken from [34] in the provided procedure. The literature [22] also described that the state and variance for multi-ahead prediction are estimated by the observation until n (Y(n)). This is not our own idea, but is used as a general assumption for multistep-ahead prediction using the linear Kalman filter. We have added the explanation and references in lines 199 - 227.

My reading of the current manuscript is that it remains unclear on several methodological points. While I acknowledge that state space models and the Kalman filter are complex issues that are hard to explain in clear detail, it just makes it more important to be clear and concise. Worse, the authors seem unwilling to revise their work for clarity and provide an honest account of their revisions.

We apologize for previous unclear explanation. We have added more details to clarify the state space representation and the Kalman filter algorithm for FO detection lines in 136 - 227.

Reviewer #2: As I wrote in my previous review, my opinion on this paper is from the viewpoint of a person with expertise in data processing, and not on biological/ecological applications.

The statistical methodology we used in this study is not novel. As the application using existing statistical methods, we newly provide the FO analysis, which is a practical approach in the Integrated ecosystem assessment for marine resource. Therefore, this paper is about biological/ecological time series application.

My opinion is that the revised version of the paper has not improved the clarity in the exposition and justification of the proposed method.

We have revised the paper carefully, especially the previous procedure for assessing unexpected tendencies.

My major criticisms concern the section "Assessing unexpected tendencies - joint probability for detected FO", starting at line 184 of the manuscript.

In this section (lines 187-188) the Authors say that the location of the true observations with respect to the predicted trend "could be" the result of a random variation. My point is that of course such locations are the result of a random variation, otherwise the process would be deterministic! The Authors should better characterize the type of randomness that they have in mind. It is a matter of what type of "random model" the future observations are more likely to be drawn from. It is a classical "Hypothesis Testing" problem in statistics.
It is not clear what are the joint probabilities that are estimated by the algorithm from line 195 to line 204.

We apologize for previous unclear explanation. We have revised the previous procedure for the section in lines 228-259 and have made the outline of the procedure in Fig.1b.

The given explanation (lines 191 to 193) is not at all clear.

We have excluded the explanation.

The Authors shoud give a more formal and rigorous definition of the probabilities p_j that are being estimated by the computations at line 199 (step 2 of the algorithm): what are the events captured by such probabilities? Moreover, what are the FO_{n+j}?

We have revised the description to obtain p_j in lines 228-259.

Next, the Authors should also give an exact defintion of the joint probability P(p_1,...,p_J) and justify the reason why the events described by the probabilities p_j are independent.

Without a formal definition it is difficult to understand if the estimation procedure is correct, or at least acceptable.

As you pointed out, we didn’t explain the independent condition to calculate the joint probability. When the observations locate above the predicted trends at e.g. three time points, for calculating the probability for each time point, the residual obtained by subtracting trend (time-dependent model) from the observation is assumed the variable that obeys identical and independent (Gaussian) distribution. Using the residual value for each time point, the upper-tale probability of the value is calculated by a computational function using Gaussian distribution function. The upper-tale probabilities $P_{1},P_{2},\text{ and }P_{3}$are independent.

Another point which is not at all discussed, is about the threshold to be considered on the estimated joint probability: when this estimated probability is to be considered so small to flag the observations?

The revised procedure applies an arbitrary threshold 0.05 which means that the event occurs by 5%-tail probability (found in the tail of the distribution). If j = 3, $P_{1}\times P_{2}\times\text{ }P_{3}$is assessed if$P_{1}\times P_{2}\times\text{ }P_{3}\leq0.05^{3}$.

Moreover, as I pointed out in my previous review, since all variables are assumed Gaussian, apparently there is no need to resort to a numerical Monte Carlo procedure, since all probabilities can be accurately computed.
The answer that the Authors gave to this issue is unclear and not satisfactory. A sequence of observations of a Gaussian process is a Gaussian vector. Since the means and covariances are known, all probabilities can be exactly computed.

The previous description was to manually calculate upper or lower tale probability using random number generated by a Gaussian distribution function. As you pointed out, it is not necessary to use the procedure and we should use the computational function to directly obtain upper or lower tale probability of the Gaussian distribution. We have revised the description in lines 228-259.

As a final remark on this section, in my opinion the right methodological framework for the problem investigated by the authors is the Statistical Hypothesis Testing. Indeed, the problem stated by the Authors consists in quantitatively assessing whether the recent observations are in accordance with the estimated past model (Null Hypothesis) or not (Alternative Hypotesis).

As you suggested, the procedure is not related to statistical testing considering null hypothesis. It is just calculation of probability based on the Gaussian distribution function. We have excluded ‘probability value’ that means significant probability to assess the test statistics.

Other issues

Lines 155-157: The authors write: "Furthermore, it is possible to identify the optimum Q and R using a numerical optimization based on the log-likelihood function (6)". Thus, it is not clear if they use or not this identification procedure or not (they only say: "is it possible")

In this paper, the variance of the system noise in Q is found by grid search within a range and the variance of the observation noise in R is given by the observation until the year before the year for multi-ahead prediction. On the other hand, there is another way to identify these variances by the numerical optimization procedure (e.g. Newton-Raphson method introduced in [34]). To avoid misunderstanding, we have modified the sentence in lines 181-183. We have also added the program using the numerical optimization procedure in Supplementary file S2.

Line 159: The section "FO analysis by multistep-ahead prediction values" is not well written.
I understand that observations are available until time n, and the state prediction for the following times is needed.
However, it is not clear the reason why it is formally assumed that Y(n)=Y(n+1)=...=Y(n+j) when j-ahead prediction has to be computed.
When observations are not available, the Kalman filter considers only the computation of the prediction step, without the correction step, and therefore no "formal" output is needed. It would be wrong to compute the Kalman correction step using past values of the observations.
Indeed, the Authors correctly compute the state predictions using the equations at line 170, without the use of any "fake" observation.

Since the future observation y(n+1) is not observed, the calculation is formally conducted by *assuming* approximately Y(n+1)=Y(n), taken from [34] in the provided procedure. The literature [22] also described that the state and variance for multi-ahead prediction are estimated by the observation until n (Y(n)). Z(n+1|n) is calculated by z(n|n) and z(n+2|n) is calculated by z(n+1|n) according to the equations at line 210. This is not our own idea, is used a general assumption for multistep-ahead prediction using the linear Kalman filter. We have added the explanation and references in lines 199 - 227.

Lines 171-174 can be improved. The Authors do not clearly distinguish between the true observation y(n+j) (unavailable) and its prediction y(n+j|n) (computable). The expectation at line 171 is indeed the prediction of the observation at n+j. The covariance at line 172 should be replaced with the error covariance Cov(y(n+j)-y(n+j|n)|Y(n)).

No, the covariance is for the distribution of y(n+j), not for the prediction error (observation error). This notation is referred in [34]. We have added the explanation to avoid misunderstanding in lines 212 - 217.

Typos:

Line 119, Either H is a row vector or a transpose symbol is missing in eq. (3)

H is a row vector if *d = 2* and is a scaler if *d = 1*. We have added the example in lines 131 - 135.

Line 204. The upper limit of the summation should be M, and not 1000.

We have not included the description anymore.

Line 230. Maybe 2013 should be 2014.

Thank you very much. We have modified it.
