# Supplementary material for: Flagged observation analyses as a tool for scoping and communication in integrated ecosystem assessments
Source: PLoS One. 2024 Sep 23;19(9):e0305716. doi: 10.1371/journal.pone.0305716 (PMC11419343; doi:10.1371/journal.pone.0305716)
Supplement: Attachment — Submitted filename: Reply to reviewers comments third.docx [file pone.0305716.s013.docx]

Dear PLOS ONE Academic Editor, João Zambujal-Oliveira,

This document contains our response to the comments and concerns provided to us by two reviewers on our submitted manuscript:

Re: PONE-D-22-30034R2

Solvang and Arneberg: Flagged observation analyses as a tool for scoping and communication in Integrated Ecosystem Assessments

The comments from the two reviewers are shown in black font, while our responses are given in red font. In the manuscript, all changes are shown using the “Track Changes” function of MS Word.

Reviewer #1

The manuscript has been revised heavily, mainly in the methodological part,

and is now more aligned with standard expositions of the Kalman filter and related

algorithms. I still find think the presentation can be written better and be more accessible

given that it is an established method. The paper should be read by a copy editor to

improve the overall writing and the odd typo.

We have gone through the manuscript carefully to improve the overall writing.

I will not comment in great detail on this

section now, the authors have revised in accordance with my earlier comments and

comments from the evidently more qualified other reviewer.

I have one comment on an issue that has been discussed earlier, and one question about the

relevant probability threshold.

The earlier discussed issue is the case with 'approximately Y(n+1) = Y(n)'. I still find this a

strange way of formulating what the authors are doing. I think what they do is to calculate

forecasted values over several time steps conditional on Y(n). That is, the forecast for n+2

is conditional on Y(n), and not on Y(n+1) with approximately Y(n+1)=Y(n). As said in

earlier correspondence, this may mainly be a notational issue, but the way the authors

states it in the present version is cumbersome and could perhaps be wrongly interpreted. I

see that the other reviewer also comments on this issue, and importantly points out that no

Kalman correction or update should be made without new observations available. This is

the kind of misunderstanding that could occur with the authors' notation.

The expression ‘assuming approximately $Y(n+1)\approx Y(n)$’ may lead to misunderstandings. What we meant is that:

Consider the case that $I\left( j \right)$ is as a set of ***actual*** observed time points in the time points $1,2,\cdots,j$, $Y\left( j \right)\equiv\left\{ y\left( i \right)|i\in I(j) \right\}.$

For one-ahead prediction, the state $z(n)$ and the variance $v(n)$ are then obtained by the Kalman filter using actual observed data series $Y\left( n \right)= \left\{ y\left( 1 \right), y\left( 2 \right),\cdots,y(n) \right\},$ that is, $z\left( n+1 | n \right)=Fz(n|n)$ and $v\left( n+1|n \right)= Fv\left( n | n \right)F^{'}+GQG'.$ Here, since the future observation $y(n+1)$ is unavailable, $Y\left( n+1 \right)= \left\{ y\left( 1 \right), y\left( 2 \right),\cdots,y\left( n \right) \right\}=Y\left( n \right).$

For long-term prediction, we consider $j$-ahead prediction of the state, $z(n+j)$, based on $Y\left( n+j \right)$, that is, $z\left( n+j | n+j \right)=Fz(n+j-1|n+j-1)$ and $v\left( n+j|n+j-1 \right)= Fv\left( n+j-1 | n+j-1 \right)F^{'}+GQG'.$

Since $y\left( n+1 \right),y\left( n+2 \right),\cdots,y(n+j)$ are not available after observed $y(n)$, $Y\left( n+j \right)= \left\{ y\left( 1 \right), y\left( 2 \right),\cdots,y\left( n \right) \right\}=Y\left( n \right).$

Therefore, the state and variance for $j$-ahead prediction are $z\left( n+j | n \right)=Fz(n+j-1|n)$ and $v\left( n+j|n \right)= Fv\left( n+j-1 | n \right)F^{'}+GQG'.$

The point is that $j$-ahead prediction based on the observations until $y(n)$ is performed by repeating the prediction step $j$ times, using the relation that $Y\left( n \right)= Y\left( n+1 \right)=\cdots=Y\left( n+j \right).$ This was from references [22, 34], and is not our own idea.

To avoid a wrong interpretation, we have rephrased the paragraph by using appropriate sentences and references and not using the notation that could lead to misunderstandings (lines 193 - 199).

I also wonder about the probability threshold that is used. For predictions over three time

steps, the authors compare with the threshold 0.05^3, which is a very small number. Thus,

at least given an approximately correct prediction model, very few observations will be flagged. And maybe this is the whole point, if the prediction model is approximately correct, there should be a small chance for flagging observations (false positives). Nevertheless, some discussion along these lines would be valuable, I think.

This threshold value is used to assess the relevance of the *p*_ut_ estimates, that is, the probability that the combined evidence of all recent observations indicates that there is an unexpected tendency across all of the most recent (i.e., predicted) years. Therefore, this threshold is not used to assess observations for single years. We agree that using the general formula for a threshold at the 0.05 level for *j* predicted years as 0.05*^j^* may give conservative estimates of what should be considered unexpected tendencies across all recent years. We have added a paragraph in the discussion and thank the reviewer for pointing out this issue (lines 407 - 413).

Reviewer #3

A thorough research has been carried out, which has applied significance.

However, some author's assumptions should be given broader explanations, which are

given in the review.

Ecosystems influence the existing conditions of human life, either improve or worsen it, and accordingly require a person's constant adaptation to such changes. Thanks to observations, it is possible to achieve the necessary predictability of ecosystems, reliably forecast and satisfy needs while under its influence. All this confirms the relevance of the article, and despite its significant advantages, it is appropriate to take into account a number of recommendations:

- the article should indicate the objectives of the observations with the overall goal of determining the scope and communications in ecosystem assessments and the expected results from such observations;

A summary of the objectives for inclusion of each of the time series in the work of the ICES integrated ecosystem assessment working group for the Norwegian Sea (WGINOR) has been added to Supplementary Table 1 (see lines 249 - 251in the main text). The objective behind the AMO data should be evident from the main text (i.e., it is described as a pronounced signal of climate variability, line 257 - 258).

- it is worth citing the advantages and disadvantages of conducting the analysis of observations with labels, in particular, comparing them with some frequently used statistical methods;

The meaning of the term «label», as used here, is not clear to us. As this could not be clarified after contact with the editor, we are unfortunately not able to address this comment.

- it is necessary to develop a set of proposals for the active implementation of the observation analysis tool with labels, addressing them to international institutions, associations of countries or others.

Again, as the meaning of the term «label», as used here, is not clear to us, we are unable to also address this comment.
