# Supplementary material for: Flagged observation analyses as a tool for scoping and communication in integrated ecosystem assessments
Source: PLoS One. 2024 Sep 23;19(9):e0305716. doi: 10.1371/journal.pone.0305716 (PMC11419343; doi:10.1371/journal.pone.0305716)
Supplement: Attachment — Submitted filename: Reply to reviewers comments forth.docx [file pone.0305716.s014.docx]

Dear PLOS ONE Academic Editor, João Zambujal-Oliveira,

This document contains our response to the comments and concerns provided to us by two reviewers on our submitted manuscript:

Re: PONE-D-22-30034R2

Solvang and Arneberg: Flagged observation analyses as a tool for scoping and communication in Integrated Ecosystem Assessments

The comments from the two reviewers are shown in black font, while our responses are given in red font. In the manuscript, all changes are shown using the “Track Changes” function of MS Word.

Reviewer #1

The manuscript looks pretty good. I suspect a remaining typo: An 'L' appears in some of the math where one may expect dots. See for example lines 136, 146, 147, and further on. May be some autocorrect playing games. It seems to have carried over from earlier versions if the track-changes version is to be believed. If so, I apologize for not spotting this earlier.

This error was introduced when our word file with the manuscript was converted to a pdf file during the submission process. We have identified this as a problem with an old version of the editor used for equations and will see to that the problem is fixed when resubmitting.

Reviewer #3

I thank the authors for considering the recommendation and wish them further scientific growth.

Regarding the concept of "labels", we mean "Flagged"

- it is worth citing the advantages and disadvantages of conducting the analysis of observations with (labels) flagged, in particular, comparing them with some frequently used statistical methods;

We thank the reviewer for clarifying his comments. One of the most common approaches to address change in time series is by using plots and analyses of anomalies. In line 409-423 and Supplementary Fig. 1, we discuss how FO analyses may give useful additional information to that held by analyses of anomalies. In addition, it has recently been shown that commonly used methods for trend detection based on frequentist approaches may produce erroneous results (see ref 54). We point out that the type of structural time series model used by us is not based on a frequentist framework but corresponds to a Bayesian approach in the state space representation and Kalman filter algorithm, which may properly assess trends in short time series (lines 383-391). We therefore think we have covered the two main advantages over other relevant frequently used statistical methods. We see no disadvantages of FO analyses when compared with the above mentioned frequently used methods, but emphasise that for analyses of anomalies, FO analyses represents a supplement rather than a replacement (lines 419-423).

- it is necessary to develop a set of proposals for the active implementation of the observation analysis tool with (labels) flagged, addressing them to international institutions, associations of countries or others.

FO analysis is primarily developed as a tool for scoping in IEA groups (see lines 50-54, 79-80 and 377-378). Thus, the primary uses of the results are the IEA groups themselves. We also point out that the results from FO analyses can be used in communication with stakeholders and that this can also be relevant for policymaking processes (lines 424-430). However, how this should be done in practice, will, in our view, be context dependent and has to be developed by IEA groups and/or users of their results. While we agree that it is highly interesting to discuss this, we feel it is beyond the scope of this manuscript, as it would mean expanding the text considerably by for example including considerable literature on the science-policy interface.
